# Effects of super high-flux vitamin E–coated and medium cut-off dialyzers on uremic toxins removal and biocompatibility: the E-FLUX randomized controlled study

**DOI:** 10.1093/ckj/sfaf106

**Published:** 2025-04-11

**Authors:** Mohamed Belmouaz, Etienne Cogne, Florent Joly, Fabien Duthe, Estelle Desport, Cecile Martin, Thierry Hauet, Sebastien Giraud, Estelle Lemarie, Lisa Durocher, Frank Bridoux

**Affiliations:** Department of Nephrology, Poitiers University Hospital, Poitiers, France; Department of Nephrology, Poitiers University Hospital, Poitiers, France; Department of Nephrology, Poitiers University Hospital, Poitiers, France; Department of Nephrology, Poitiers University Hospital, Poitiers, France; Department of Nephrology, Poitiers University Hospital, Poitiers, France; Department of Nephrology, Poitiers University Hospital, Poitiers, France; Department of Pharmacology, Poitiers University Hospital, Poitiers, France; Institut National de la Sante et de la Recherche Médicale (INSERM) IRMETIST U1313, Poitiers University Hospital, Poitiers, France; Institut National de la Sante et de la Recherche Médicale (INSERM) IRMETIST U1313, Poitiers University Hospital, Poitiers, France; Department of Biostatistics, Centre d'Investigation Clinique CIC 1402, Poitiers University Hospital, Poitiers, France; Department of Nephrology, Poitiers University Hospital, Poitiers, France

**Keywords:** beta2-microglobulin, biocompatibility, medium cut-off dialyzer, oxidative stress, vitamin E-coated super high-flux dialyzer

## Abstract

**Background:**

Medium cut-off hemodialysis (MCO-HD) improves removal of middle molecules (MM) uremic toxins. The effects of the novel super high-flux vitamin E–coated (SHFVE) dialyzer on MM removal and biocompatibility parameters including inflammation and oxidative stress remain to be investigated.

**Methods:**

This non-inferiority cross-over prospective randomized study included 36 patients randomly assigned to receive either 3 months of MCO-HD followed by 3 months of SHFVE-HD, or vice versa. The primary endpoint was beta2-microglobulin reduction ratio (RR) after 3 months. Secondary endpoints were other MM RR and biocompatibility parameters.

**Results:**

SHFVE-HD provided non-inferior beta2-microglobulin RR as compared with MCO-HD {74.2% [95% confidence interval (CI) 71; 77] vs 73.3% (95% CI 71; 76) with a difference of 0.9% (95% CI –1.9%; 3.6), respectively}, with similar mean RR of prolactin, alpha1-microglobulin, vascular endothelial growth factor, and kappa and lambda free light chains. SHFVE-HD induced lower mean myoglobin RR compared with MCO-HD (55.5 ± 7.3 vs 60.2 ± 6.6%, *P *= 0.022). Myoglobin pre-dialysis levels were not significantly different [160 (118–199) vs 167 (167–240) µg/L, *P *= .08].

Median pre-dialysis levels of interleukin-6 [0.8 (0–4.4) vs 1.7 (0.2–7.2) pg/mL, *P *= .032], asymmetric dimethylarginine (ADMA) [163 (122–260) vs 167 (133–270) ng/mL, *P *= .01), mean pre-dialysis serum soluble tumor necrosis factor receptor 1 (sTNFR1) levels (12.7 ± 3.5 vs 13.6 ± 3.6 ng/mL, *P *= .039) and mean post-dialysis oxidized low-density lipoprotein levels (54 ± 18 vs 63 ± 22 ng/mL, *P *= .01) decreased significantly with SHFVE-HD. SHFVE-HD induced a significantly lower median relative variation in blood leucocyte count 15 min after dialysis initiation [–3.5 (–6.8 to 1.6) vs –6.2 (–12.9 to –1.5) %, *P *= .009], encompassing both polymorphonuclear neutrophils and monocytes.

**Conclusion:**

Compared with MCO-HD, SHFVE-HD appears to provide similar MM RR and may be associated with improved biocompatibility parameters.

**Trial registration: ClinicalTrials.gov:**

NCT05610683. Data deposited at Centre de la recherche clinique CHU Poitiers

## Graphical Abstract

**Graphical Abstract:**
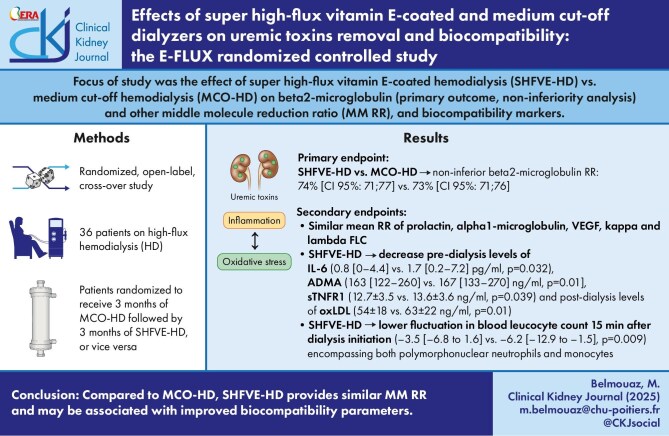

KEY LEARNING POINTS **What was known:**

- Hemodialysis patients are exposed to the accumulation of uremic toxins and to bioincompatibility induced by blood and dialyzer interaction.
- Vitamin E–coated dialyzers have potential beneficial effects on biocompatibility.
- Super high-flux vitamin E–coated (SHFVE) dialyzers could provide similar efficient removal of uremic toxins to medium cut-off dialyzers, with improved biocompatibility profile.

**This study adds:**

- This study confirms the non-inferiority of SHFVE dialyzers on beta2-microglobulin and other middle molecules reduction ratios, as compared with medium cut-off dialyzers.
- In addition, SHFVE dialyzers was associated with improved biocompatibility markers, including interleukin-6, serum soluble tumor necrosis factor receptor 1 (sTNFR1), asymmetric dimethylarginine (ADMA) and oxidized low-density lipoprotein, and reduced variation of blood leucocyte count.

**Potential impact:**

- The efficient removal of middle molecule uremic toxins along with better biocompatibility profile of SHFVE dialyzers suggests potential benefits on morbidity and mortality in hemodialysis patients.
- Large prospective studies are required to confirm the effects of this strategy on long-term clinical outcomes.

## INTRODUCTION

Patients on maintenance hemodialysis (HD) are exposed to long-term accumulation of uremic toxins, excessive oxidative stress and inflammation, contributing to increased morbidity and mortality [1–3].

Uremic toxins cover a wide range of molecules, classified according to their molecular weight as small-size (<500 Da), middle-size (500 Da–60 kDa) and protein-bound toxins [4]. Over the past decades, low-flux HD has been the most commonly used dialysis technique, providing effective clearance of small solutes, but negligible removal of middle molecules (MM). This limitation was partially overcome with the development of high-flux (HF) dialyzers, characterized by a cut-off pore size of 15–20 kDa. Subsequently, HF dialyzers used in post-dilution on-line hemodiafiltration (OL-HDF) mode demonstrated effective clearance of MM [5]. Simultaneously, specific super high-flux (SHF) dialyzers integrating higher cut-off pore size were developed in Japan and classified as type V dialyzers, according to beta2-microglobulin clearance ≥70 mL/min [6]. Type V SHF dialyzers, used in HD mode (SHF-HD) and the medium cut-off (MCO) dialyzer (Theranova™), with higher and controlled porosity resulting in a steep sieving curve and cut-off value of 42 kDa, have demonstrated similar MM depuration. These dialyzers have been shown to improve MM depuration compared with HF-HD and to provide similar MM removal compared with OL-HDF [7–13].

Apart from uremic toxins, oxidative stress and inflammation in HD is generated by a variety of conditions, including dialysate purity, vitamin deficiency, iron administration, vascular access and dialysis procedure. Dialyzer biocompatibility depends on physicochemical characteristics of the material that affects the release of pro-inflammatory cytokines and reactive oxygen species (ROS) induced by the long-term intradialytic contact with blood [14]. Based on the potent hydrophilic scavenging antioxidant capacity of vitamin E to protect plasma lipids and cell membranes from peroxidation [15], vitamin E–coated bioactive dialyzers have been developed and showed reduced HD-related oxidative stress and inflammation [16]. However, the effects of the novel vitamin E–coated SHF (SHVE) dialyzer have not been investigated yet.

The aim of the present study was to compare the efficacy of the type V super high-flux vitamin E–coated (SHFVE) dialyzer ViE™-X (Asahi Kasei Medical Co. Ltd, Tokyo, Japan) versus the MCO dialyzer Theranova™ 500 (Baxter Healthcare Corporation, Deerfield, IL, USA) on the removal of beta2-microglobulin and other MM in a non-inferiority analysis, and their respective effects on biocompatibility markers.

## MATERIALS AND METHODS

### Study design

This randomized, open-label, cross-over design study (ClinicalTrials.gov: NCT05610683) was conducted in accordance with the ethical principles of the Declaration of Helsinki and approved by local ethics committees (Comité de Protection des Personnes ILE DE FRANCE X). After informed written consent, patients were randomized to receive 3 months of MCO-HD followed by 3 months of SHFVE-HD, or vice versa (Table 1). At 3 months from randomization, each patient underwent a 2-week HF-HD washout period using the Elisio™ 21H dialyzer (Nipro Europe, Zaventem, Belgium) prior to crossover. Random allocation was generated electronically. The trial was not blinded either to patients or to physicians.

**Table 1:** Characteristics of dialyzers.

Dialyzer	Membrane polymer	Membrane type	Fiber length (mm)	Fiber inner diameter (μm)	Membrane area (m²)	Membrane wall thickness (μm)	UFC (mL/h/mmHg)	Beta2-m SC	Albumin SC	Sterilization
ViE™-X	Vitamin E-interactive polysulfone	SHFVE	266	185	2.1	45	104	0.9	<0.01	Gamma-Ray
Theranova™ 500	PAES/PVP	MCO	236	180	2	35	59	1	0.008	Steam

PAES/PVP, polyarylethersulfone/polyvinylpyrrolidone; UFC, ultrafiltration coefficient; Beta2-m, beta2-microglobulin; SC, sieving coefficient.

### Patient selection

Chronic HD patients were enrolled at the HD unit of CHU Poitiers between June 2023 and July 2023. The study ended on 30 January 2024.

The inclusion criteria were as follows: (i) patients aged >18 years; (ii) receiving thrice weekly maintenance HD for >6 months, using a high-flux dialyzer (Supplementary data, Table S1) for >3 months; (iii) with permanent vascular access (fistula or tunneled central venous catheter); and (iiii) able to give written informed consent. Exclusion criteria were uncontrolled cancer, any ongoing condition that may interfere with inflammatory parameters, baseline C-reactive protein (CRP) >40 mg/L, pregnant or breast-feeding women, any medical condition, psychiatric disorder or biological abnormality that might interfere with subject's participation or ability to sign an informed consent form.

### Dialysis modalities

All patients received thrice weekly HD throughout the trial with the following modalities: 240 min session using a Nikkiso DBB-EXA (Hemotech Ramonville Saint Agne, France) or an Artis (Baxter Healthcare Corporation, Deerfield, IL, USA) console, bicarbonate dialysis, citrate-containing acetate-free ultrapure dialysate, dialysate flow at 500 mL/min, targeted mean blood flow >300 mL/min and systematic anticoagulation with enoxaparin.

Dialysis parameters including type of dialyzer, blood flow rate, session length, body weight before and after treatment, total ultrafiltration, online Kt and Kt/V through ionic dialysance or UV-absorbance were recorded at selected dialysis sessions using the Hemabox^®^ system and collected on the Hemadialyse^®^ (Gambro Dialysatoren GmbH, Hechingen, Germany) database.

### Sample collection and laboratory analysis

Pre-dialysis, 15 min after initiation and post-dialysis blood samples were taken on the day of inclusion, at 3 months post-inclusion, and at 3 months after the cross-over phase. Blood samples were collected from the arterial blood line at mid-week dialysis session. The slow-flow method was used for post-dialysis blood samples collection.

Routine parameters were measured locally. Additional tests were performed on pre-dialysis, 15 min after initiation and post-dialysis serum samples obtained after immediate centrifugation for 10 min and stored at –80ºC (Supplementary Methods).

### Calculations

Reduction ratios (RR) were calculated as follows: RR = [(Cpre – Cpost)/Cpre], where Cpre and Cpost are the pre- and post-treatment concentrations, respectively. Post-treatment concentrations of MM were corrected for hemoconcentration using a single-compartment kinetic model [17]. Serum levels of inflammation and oxidative stress markers, and leucocyte/platelet counts measured at 15 min and after dialysis were corrected for hematocrit concentration [18] (Supplementary Methods). Relative variation (RV) of leucocyte and platelet was calculated as follows: [(Cfinal *–* Cinitial)/Cinitial], where Cfinal are the 15 min after dialysis or post-dialysis values and Cinitial is the pre-dialysis value, respectively.

Single-pool KT/V (spKT/V) and normalized protein catabolic rate (nPCR) were calculated according to the Daugirdas formula [19] and Depner equation [20], respectively.

Erythropoietin resistance index (ERI) was defined as the weekly dose of erythropoietin-stimulating agents (ESA) per body weight divided by hemoglobin level (Supplementary Methods).

### Endpoints

The primary endpoint was the noninferiority of beta2-microglobulin RR after 3 months of SHFVE-HD versus MCO-HD. Beta2-microglobulin RR was chosen because this routinely measured MM (12 kDa) uremic toxin is an independent mortality risk factor in HD [21].

Secondary endpoints were removal of small molecules, other MM RR and exploratory parameters including pre-dialysis, 15 min and post-dialysis concentrations biocompatibility markers.

### Sample size calculation

A non-inferiority margin of 8% was defined for the primary endpoint, based on the results of a small previous study [22]. To demonstrate the non-inferiority of beta2-microglobulin RR between SHFVE-HD and MCO-HD arm, with an alpha risk of 2.5% and a statistical power of 80%, a total of 32 patients was required. Assuming a dropout rate of 20%, 38 patients were recruited.

### Statistical analysis

**Figure 1:**
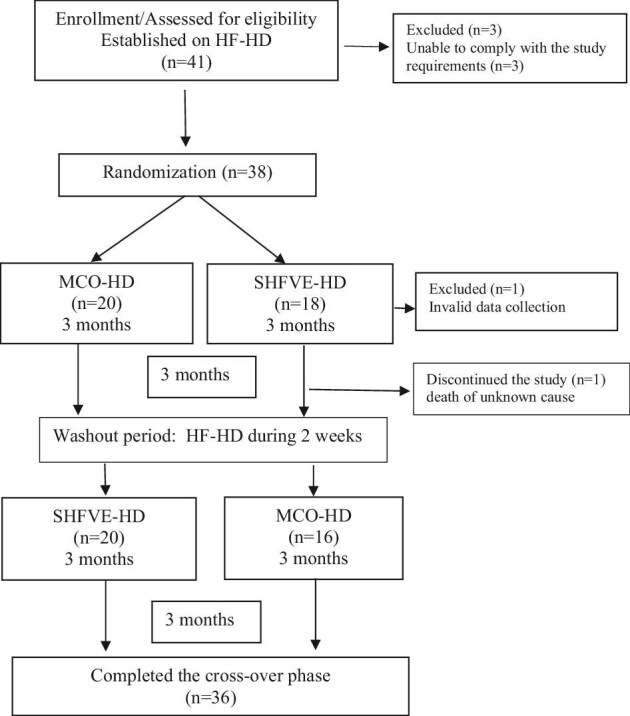
Patient flow chart.

Analyses were performed on a modified intention-to-treat basis. The modified intention to treat population consisted of 36 patients who completed the cross-over study. One patient with invalid data collection and one patient who died during the SHFVE-HD period before complete sample collection were not analyzed (Fig. 1). Continuous data were expressed as means ± standard deviation (SD) or median [interquartile range (IQR)]. Categorial variables are given as number (percentage) of patients.

For the primary endpoint, the RR of beta2-microglobulin and its 95% confidence intervals (95% CIs) were estimated using a mixed model with fixed effects of period and study dialyzer type, and the random effect of subject. The secondary endpoints were compared using the same mixed model. To validate the models created, the hypotheses of homoscedasticity and the normality of the residuals were verified. If the hypotheses were not validated, we performed a cubic root transformation on the criteria. All hypotheses were tested at the 5% significance level. Analyses were performed using the R software package, version 4.0.4.

## RESULTS

The crossover study population comprised 20 patients assigned to receive 3 months of MCO-HD followed by 3 months of SHFVE-HD, and 16 patients assigned to receive 3 months of SHFVE-HD followed by 3 months of MCO-HD. Each patient received a 2-week washout period using HF-HD after 3 months (Fig. 1).

### Patient baseline characteristics

Twenty-five of the 36 patients had negligible renal residual function with urine output <200 mL/day, and 11 had a residual urine output between 300 and 500 mL/day (Table 2). The mean age was 69.8 ± 13.6 years, and 39% of patients were dialyzed with a permanent tunneled catheter. Dialysis-related parameters on SHFVE-HD and MCO-HD after 3 months are shown in Table 3. Blood flow, dialysate flow, ultrafiltration volume, session length, processed blood volume, and Kt and Kt/V monitor values were similar in both groups.

**Table 2:** Baseline characteristics of the study population.

	All (*n* = 36)	SHFVE-HD first (*n* = 16)	MCO-HD first (*n* = 20)
Age (years)	69.8 ± 13.6	70 ± 11.9	69.7 ± 15.2
Gender (female/male), *n* (%)	17 (47)/19 (53)	7 (44)/9 (56)	10 (50)/10 (50)
Body height (cm)	165 ± 9.1	162 ± 6.1	167 ± 10.4
Body weight (kg)	72.9.5 ± 18	70 ± 18.5	75.2 ± 17.7
BMI (kg/m²)	26.7 ± 6	26.6 ± 6.6	26.8 ± 5.7
Dialysis vintage (years)	2.7 (1.7–7.2)	2.2 (1.5–6.9)	3.2 (1.7–7.2)
Residual urine output, *n* (%)			
<200 mL/day	25 (69)	10 (62)	15 (75)
>300 and <500 mL/day	11 (31)	6 (38)	5 (25)
CKD cause, *n* (%)			
Diabetic nephropathy	3 (8)	1 (6)	2 (10)
Glomerulopathy	5 (14)	2 (12)	3 (15)
Interstitial nephritis	3 (8)	1 (6)	2 (10)
Nephrosclerosis	9 (25)	3 (19)	6 (30)
Polycystic kidney disease	5 (14)	4 (25)	1 (5)
Unknown	11 (31)	5 (31)	6 (30)
Comorbidities, *n* (%)			
Diabetes	12 (33)	5 (31)	7 (35)
Hypertension	31 (86)	14 (88)	17 (85)
Dyslipidemia	18 (50)	6 (38)	12 (60)
Heart disease	4 (11)	0 (0)	4 (20)
Cerebrovascular disease	4 (11)	1 (6)	3 (15)
Peripheral vascular disease	8 (22)	4 (25)	4 (20)
Vascular access, *n* (%)			
Native arteriovenous fistula	22 (61)	12 (75)	10 (50)
Permanent tunneled catheter	14 (39)	4 (25)	10 (50)
Blood flow rate (mL/min)	300 (300–337)	300 (300–350)	300 (300–337)
Dialysate flow rate (mL/min)	500	500	500
Session length (min)	237 ± 9	237 ± 7	238 ± 10
Blood volume processed (L)	72 (68–78)	72 (68–72)	74 (72–78)
Ultrafiltration (L)	2.1 ± 0.7	2 ± 0.7	2.2 ± 0.8
Kt (mL/min)^b^	221 ± 38	209 ± 34	231 ± 39
Kt/V monitor^a^	1.4 ± 0.3	1.4 ± 0.3	1.4 ± 0.2

^a^One missing data.

^b^three missing data.

Quantitative data are expressed as mean ± SD or median (IQR).

BMI, body mass index; CKD, chronic kidney disease.

**Table 3:** Dialysis parameters after 3 months of SHFVE-HD versus MCO-HD.

	SHFVE-HD	MCO-HD	*P*-value
Blood flow rate (mL/min)	306 ± 29	304 ± 32	.71
Dialysate flow rate (mL/min)	500	500	NS
Session length (min)	237 ± 10	238 ± 8	.75
Blood volume processed (L)	69 ± 8	68 ± 10	.5
Ultrafiltration (L)	1.9 ± 0.7	2.1 ± 0.8	.49
Kt (mL/min)^a^	222 ± 39	221 ± 36	.88
Kt/V monitor^a^	1.4 ± 0.3	1.4 ± 0.3	.70

Quantitative data are expressed as mean ± SD.

^a^One missing data.

Serum concentration of uremic toxins levels, inflammatory and oxidative stress parameters, routine biological parameters, leucocyte and platelet counts at randomization are reported in the Supplementary data, Tables S2–S5

### Primary endpoint

The beta2-microglobulin RR achieved after 3 months of SHFVE-HD was found not inferior to that with MCO-HD: 74.2% (95% CI 72.1; 76.4) and 73.3% (95% CI 71.2; 75.5), respectively. The difference between the two dialyzers was 0.9% (95% CI –1.8; 3.6), which is above the non-inferiority margin set at 8%.

### Secondary endpoints

#### Routine biological parameters

No significant difference was observed regarding nPCR calculation, albumin, transthyretin, CRP and serum amyloid A (SAA) levels (Table 4). The mean hemoglobin level did not differ between SHFVE-HD and MCO-HD (12 ± 1.4 g/dL vs 11.5 ± 1.4, *P *= .1). Parameters of iron transport and metabolism, ERI, use of iron and ESA dose during SHFVE-HD and MCO-HD period were similar (Table 5).

**Table 4:** Routine biological parameters after 3 months of SHFVE-HD versus MCO-HD.

	SHFVE-HD	MCO-HD	*P*-value
Albumin (g/L)			
Pre-dialysis levels	37.7 ± 4.1	37 ± 3.7	.63
Post-dialysis levels	41.1 ± 4.0	41.2 ± 4.9	.95
Transthyretin (g/L)	0.3 ± 0.1	0.3 ± 0.1	.79
nPCR	1 ± 0.2	1 ± 0.3	.44
CRP (mg/L)	4 (2–18)	6 (2–17)	.47
SAA (mg/L)	9.8 (5–35)	12.5 (5–26)	.87
Hemoglobin (g/dL)	12 ± 1.4	11.5 ± 1.4	.1
Ferritin (µg/L)	468 (226–671)	490 (358–683)	.18
Transferin saturation index (%)	24 ± 9.7	27.5 ± 13.8	.10
Ret-he (pg)	32.3 ± 4	32.5 ± 3.5	.93
LDH (UI/L)	202 ± 39.3	203 ± 48.8	.94

Quantitative data are expressed as mean ± SD or median (IQR).

Ret-he, hemoglobin content of reticulocytes; LDH, lactate dehydrogenase.

**Table 5:** Delivered ESA and iron treatments after 3 months of SHFVE-HD versus MCO-HD.

	SHVE-HD	MCO-HD	*P*-value
ESA, *n* (%)	32 (89)	33 (92)	1
Darbepoetin alpha	100%	100%	NS
ESA (UI/week)	28.9 (16.5–58)	30.4 (10.3–50.4)	.54
ESA (UI/12 weeks)	510 ± 491	459 ± 422	.24
Iron sucrose, *n* (%)	29 (81)	28 (78)	.77
Iron dose (mg/week)	53 (11.8–96.4)	59 (7.6–95.7)	.86
Cumulated iron dosage (mg/12 weeks)	673 ± 615	663 ± 578	.95
ERI (IU/kg/week/g/dL)	8 (3–14)	6 (3–18)	.73

Quantitative data are expressed as mean ± SD or median (IQR).

NS, not significant.

#### Removal of small solutes

RR of urea, creatinine, Kt and Kt/V monitor and spKt/V calculation did not differ significantly between SHFVE-HD and MCO-HD (Tables 3 and 6).

**Table 6:** Uremic toxins after 3 months of SHFVE-HD versus MCO-HD.

	SHFVE-HD	MCO-HD	*P*-value
Urea			
Pre-dialysis (mmol/L)	19.0 (17–21)	21.0 (16–25)	NP
Post-dialysis (mmol/L)	4.7 (3.7–5.2)	4.8 (3.5–6.3)	NP
RR (%)	76.1 ± 6.1	75.5 ± 6.8	.41
spKT/V	1.6 ± 0.3	1.5 ± 0.4	.41
Creatinine			
Pre-dialysis (μmol/L)	631 (524–762)	706 (570–815)	NP
Post-dialysis (μmol/L)	199 (162–285)	217 (148–292)	NP
RR (%)	67.1 ± 6.9	67.5 ± 6.8	.53
Beta2-microglobulin (11.8 kDa)			
Pre-dialysis (mg/L)	28.0 (24–30)	27.0 (23.5–29.5)	.14
Post-dialysis (mg/L)	6.7 (5.9–7.6)	6.4 (5.7–7.6)	NP
RR (%)	74.2 (95% CI 71.5; 76.8)	73.3 (95% CI 70.8; 75.7)	.9
Myoglobin (17 kDa)			
Pre-dialysis (µg/L)	160 (118–199)	167 (105–240)	.0813
Post-dialysis (µg/L)	69 (56–89)	65 (45–98)	.7
RR (%)	55.5 ± 7.3	60.2 ± 6.6	**.0224**
Prolactin (23 kDa)^b^			
Pre-dialysis (ng/mL)	20.7 (15–38.6)	19.4 (13.8–42.8)	.61
Post-dialysis (ng/mL)	8.5 (6.5–17.4)	8.3 (5.5–14.4)	NP
RR (%)	56.5 ± 8.9	55.9 ± 12.3	.71
Alpha1-microglobulin (30 kDa)^d^			
Pre-dialysis (µg/mL)	329 ± 90	342 ± 85	.22
Post-dialysis (ng/mL)	271 ± 72	285 ± 84	NP
RR (%)	16.7 ± 13.3	16.5 ± 14.8	.77
VEGF (43 kDa)^d^			
Pre-dialysis (pg/mL)	61.8 (28–123)	61.6 (27–121)	.78
Post-dialysis (pg/mL)	41 (15–82)	33.7 (12.9–76.3)	NP
RR (%)	35.4 ± 27.1	45.4 ± 31.1	.11
Kappa FLC (22 kDa)^a^			
Pre-dialysis (mg/L)	132 ± 46	135 ± 42	.26
Post-dialysis (mg/L)	60 ± 24	62 ± 25	NP
RR (%)	55.2 ± 9.4	55.3 ± 9.8	.9
Lambda FLC (45 kDa)^c^			
Pre-dialysis (mg/L)	106 (79–130)	102 (75–134)	.68
Post-dialysis (mg/L)	69 (54–101)	70 (51–92)	NP
RR (%)	30.8 ± 8.0	31.1 ± 8.7	.29

Quantitative data are expressed as mean ± SD or median (IQR).

^a^One missing data.

^b^two missing data.

^c^three missing data.

^d^four missing data.

NP, not performed.

Values appear in bold if P-value was significant (below 0.05).

#### Removal of middle molecules

SHFVE-HD and MCO-HD induced similar mean RR of prolactin (56.5 ± 8.9 vs 55.9 ± 12.3%, *P *= .71), alpha1-microglobulin (16.7 ± 13.3 vs 16.5 ± 14.8%, *P *= .77), vascular endothelial growth factor (VEGF) (35.4 ± 27.1 vs 45.4 ± 31.1%, *P *= .11), kappa (55.2 ± 9.4 vs 55.3 ± 9.8%, *P *= .9) and lambda (30.8±8 vs 31.1 ± 8.7%, *P *= .29) free light chains (FLC). There was no significant difference in mean or median pre-dialysis levels of these toxins and in median pre-dialysis beta2-microglobulin levels (Table 6).

Compared with SHFVE-HD, MCO-HD provided a significantly higher mean myoglobin RR (55 ± 7.3% vs 60 ± 6.6%, respectively, *P *= .022). However, we observed a non-significant trend towards lower myoglobin pre-dialysis levels with SHFVE-HD [160 (118–199) vs 167 (105–240) µg/L, respectively, *P *= .081].

#### Markers of inflammation

SHFVE-HD induced a significant decrease in median serum interleukin (IL)-6 concentration taken pre-dialysis [0.8 (0–4.4) vs 1.7 (0.2–7.2) pg/mL with MCO-HD, *P *= .032], at 15 min after dialysis initiation [0.1 (0–3.8) vs 1.2 (0.1–6.6) pg/mL, *P *= .013] and post-dialysis [0.2 (0–6.4) vs 1.6 (0.1–10.8) pg/mL, *P *= .006]. In addition, SHFVE-HD produced a significant decrease in mean serum soluble tumor necrosis factor receptor 1 (sTNFR1) (12.7 ± 3.5 vs 13.6 ± 3.6 ng/mL, *P *= .039) and a trend towards lower median serum pre-dialysis tumor necrosis factor-alpha (TNF-α) concentration (*P *= .074).

SHFVE-HD and MCO-HD achieved similar pre-dialysis, 15 min and post-dialysis hepcidin serum levels (Table 7).

**Table 7:** Inflammatory parameters and markers of oxidative stress after 3 months of SHFVE-HD versus MCO-HD.

	SHFVE-HD	MCO-HD	*P*-value
IL-6			
Pre-dialysis (pg/mL)	0.8 (0–4.4)	1.7 (0.2–7.2)	**.032**
After 15 min (pg/mL)	0.1 (0–3.8)	1.2 (0.1–6.6)	**.013**
Post-dialysis (pg/mL)	0.2 (0–6.4)	1.6 (0.1–10.8)	**.006**
TNF-α			
Pre-dialysis (pg/mL)	0.7 (0.1–6.4)	0.9 (0.1–5)	.074
After 15 min (pg/mL)	1 (0.1–4.6)	0.1 (0.1–1.8)	.39
Post-dialysis (pg/mL)	0.1 (0.1–1)	0.1 (0.1–1.7)	.21
sTNFR1			
Pre-dialysis (ng/mL)	12.7 ± 3.5	13.6 ± 3.6	**.039**
After 15 min (ng/mL)	13.4 ± 3.8	13.8 ± 3.8	.27
Post-dialysis (ng/mL)	10.1 ± 3.5	9.8 ± 2.8	.66
Hepcidin			
Pre-dialysis (ng/mL)	98.5 ± 69.9	118.6 ± 92.9	.11
After 15 min (ng/mL)	90.8 ± 67.9	111 ± 86.8	.12
Post-dialysis (ng/m)	73.5 ± 59.3	83.7 ± 58.3	.22
Ox-LDL			
Pre-dialysis (U/L)	52.5 ± 19	55.6 ± 21	.28
After 15 min (U/L)	63.7 ± 23	66.8 ± 27	.48
Post-dialysis (U/L)	53.9 ± 17.9	63.5 ± 22.6	**.016**
SOD activity^a^			
Pre-dialysis (U/mL)	3.5 ± 1.9	3.5 ± 2.4	.86
After 15 min (U/mL)	3.5 ± 1.9	4.1 ± 2.4	**.019**
Post-dialysis (U/mL)	2.3 ± 1.4	2.2 ± 1.4	.42
MDA			
Pre-dialysis (ng/mL)	153 (114–299)	179 (117–321)	.63
After 15 min (ng/mL)	176 (120–307)	173 (125–305)	.79
Post-dialysis (ng/mL)	155 (91–285)	165 (110–279)	.37
ADMA			
Pre-dialysis (ng/mL)	163 (122–260)	167 (133–270)	**.014**

Quantitative data are expressed as mean ± SD or median (IQR).

^a^Two missing data.

Values appear in bold if P-value was significant (below 0.05).

#### Markers of oxidative stress

SHFVE-HD induced a significant decrease in median serum asymmetric dimethylarginine (ADMA) pre-dialysis levels [163 (122–260) vs 167 (133–270) ng/mL, *P *= .014].

A significant decrease in post-dialysis oxidized low-density lipoprotein (ox-LDL) levels (53.9 ± 17.9 vs 63.5 ± 22.6 U/L, *P *= .016) was observed, without significant difference in mean pre-dialysis or 15 min after dialysis initiation (Table 7). We found no difference in serum pre-dialysis, 15 min after initiation and post-dialysis malondialdehyde (MDA) levels between SHFVE-HD and MCO-HD. Although mean pre- and post-dialysis concentration of superoxide dismutase (SOD) activity were comparable, a lower mean SOD activity at 15 min was observed with SHFVE-HD (3.5 ± 1.9 vs 4.1 ± 2.4 U/mL, *P *= .019).

#### Leucocyte and platelet fluctuation

SHFVE-HD induced a significantly lower leucocyte RV at 15 min [–3.5 (–6.8 to 1.6) vs –6.2 (–12.9 to –1.5), *P *= .009], as compared with MCO-HD (Table 8). Both polymorphonuclear neutrophil and monocyte counts showed significantly lower RV at 15 min [–0.4 (–5.3 to 4.0) vs –3.4 (–10.8 to –0.2), *P *= .011 and –12.8 (–21.2 to –8.1) vs –21.8 (–30.9 to –8.5), *P *= .0325]. Platelet fluctuation assessed by RV at 15 min was comparable between SHFVE-HD and MCO-HD.

**Table 8:** Leucocyte and platelet fluctuation after 3 months of SHFVE-HD versus MCO-HD.

	SHFVE-HD	MCO-HD	*P*-value
Leucocyte			
Pre-dialysis (^9^ cells/L)	6 (5.4–6.7)	6.2 (5.4–7.7)	NP
After 15 min (10^9^ cells/L)	6.1 (5–6.7)	5.7 (5.1–6.7)	NP
Post-dialysis (10^9^ cells/L)	5.9 (5–6.7)	6 (4.9–7.1)	NP
RV (%) after 15 min	–3.5 (–6.8 to 1.6)	–6.2 (–12.9 to –1.5)	**.0099**
RV (%) after 240 min	–1.6 (–13.0 to 5.5)	–3.9 (–13.1 to 9.9)	.75
Polymorphonuclear neutrophil			
Pre-dialysis (10^9^ cells/L)	4.2 ± 1.4	4.2 ± 1.4	NP
After 15 min (10^9^ cells/L)	4.3 ± 1.5	4 ± 1.4	NP
Post-dialysis (10^9^ cells/L)	4.1 ± 1.6	4.2 ± 1.7	NP
RV (%) after 15 min	–0.4 (–5.3 to 4.0)	–3.4 (–10.8 to –0.2)	**.0111**
RV (%) after 240 min	–4.3 (–12.7 to 7.2)	–3.4 (–16.2 to 14.0)	.28
Monocyte			
Pre-dialysis (10^9^ cells/L)	0.7 ± 0.2	0.7 ± 0.3	NP
After 15 min (10^9^ cells/L)	0.6 ± 0.2	0.5 ± 0.2	NP
Post-dialysis (10^9^ cells/L)	0.6 ± 0.2	0.6 ± 0.2	NP
RV (%) after 15 min	–12.8 (–21.2 to –8.1)	–21.8 (–30.9 to –8.5)	**.0325**
RV (%) after 240 min	–15.3 (–24.6 to –2.9)	–18.8 (–26.0 to 3.8)	.48
Platelet			
Pre-dialysis (10^9^ cells/L)	194 (160–234)	194 (155–225)	NP
After 15 min (10^9^ cells/L)	195 (156–220)	192 (158–217)	NP
Post-dialysis (10^9^ cells/L)	196 (169–245)	195 (158–240)	NP
RV (%) after 15 min	–1.9 (–6.5 to 1.9)	–0.9 (–6.0 to 3.6)	.78
RV (%) after 240 min	2.7 (–3.2 to 9.7)	–5.4 (–3.8 to 11.5)	.99

Quantitative data are expressed as mean ± SD or median (IQR).

NP, not performed.

Values appear in bold if P-value was significant (below 0.05).

## DISCUSSION

In this randomized study, a crossover comparison between SHFVE-HD and MCO-HD was conducted under matched conditions, including use of dialysate ultrapure fluid with similar blood and dialysate flow rates. The noninferiority of beta2-microglobulin RR (primary endpoint), a MM associated with inflammation and mortality [21] was confirmed with SHFVE-HD, as compared with MCO-HD. In addition, RR of small solutes and various MM, including prolactin (23 kDa), alpha1-microglobulin (30 kDa), VEGF (43 kDa), and kappa (22 kDa) and lambda (45 kDa) FLC, also involved in morbidity and mortality of HD patients [2, 3], were comparable. Two previous prospective studies [22, 23] also showed that SHFVE-HD has similar efficiency to MCO-HD for the removal of small and MM uremic toxins. However, our findings differed from those reported by Maduell *et al*. [23] who found similar depuration of myoglobin (17 kDa), a MM uremic toxin involved in organ damage, oxidative stress and mitochondrial dysfunction [2]. In our study, SHFVE-HD induced a significant lower myoglobin RR compared with MCO-HD. This discrepancy may be attributed to inter- and intra-individual variation in myoglobin levels among HD patients. Unexpectedly, we observed a non-significant trend towards lower pre-dialysis myoglobin levels. Further investigation is needed to determine whether this finding is due to a potential vitamin E–related antioxidant effect. In view of these results, SHFVE could be categorized as an MCO dialyzer.

Beside filtration and removal of uremic toxins, oxidative stress and inflammation both contribute to the development of cardiovascular disease and arteriosclerosis in HD patients [1]. The dialysis membrane is the main site of blood exposure to non-biological material, which physicochemical characteristics trigger various biological reactions and determine biocompatibility. Vitamin E is a lipophilic antioxidant and an effective ROS scavenger [16, 24]. Although several studies suggested a beneficial effect of vitamin E oral administration on oxidative stress, and protection against cardiovascular outcomes in a randomized clinical trial [25], the clinical benefits of this strategy remain unproven [1, 15].

Vitamin E–coated dialyzers were developed to reduce the production of ROS triggered by the contact between blood cells and the dialysis membrane, which stimulates the activation of leucocytes, platelets and red blood cells, directly or indirectly via the complement and coagulation systems [26], finally resulting in the release of inflammatory cytokines and increased oxidative stress. Of note, the antioxidative effects of vitamin E are exerted through the in-situ action of vitamin E–coated dialyzer on circulating leucocytes, rather than through its systemic release [15, 24, 27].

Our results are in line with previous published meta-analyses on vitamin E–coated dialyzers [28, 29] that showed improved IL-6 pre-dialysis levels without change in CRP level, both being considered as strong predictors of mortality [30, 31]. SHFVE-HD achieved significantly lower pre-dialysis IL-6 levels after 3 months, and improved IL-6 levels at 15 min and post-dialysis. Removal of IL-6 (24 kDa) is unlikely as the two dialyzers achieve similar MM removal. Moreover, SHFVE-HD achieved a significant reduction of sTNFR1 pre-dialysis levels, an independent predictor of mortality in HD patients [32, 33], with a non-significant trend towards lower pre-dialysis levels of the proinflammatory cytokine TNF-α after 3 months.

Hepcidin, a 10-kDa peptide, which production is increased by inflammation, contributes to the dysregulation of iron metabolism, through impaired release of stored iron. Current data on the kinetics and clearance of hepcidin in HD patients are conflicting [34, 35]. We found that both SHFVE-HD and MCO-HD induced similar pre-dialysis, 15 min and post-dialysis hepcidin levels.

Results from previous studies [36, 37] showed improved pre-dialysis levels of ox-LDL and plasma MDA, after 6 months of HD with vitamin E–coated HF polysulfone dialyzers. Ox-LDL and MDA (a final product of the peroxidation of polyunsaturated fatty acids) may promote atherosclerosis [38]. In our study, we found no difference in serum pre-dialysis levels of MDA and ox-LDL. Whether the short 3-month duration or the use of serum rather than plasma concentrations may account for these results remains to be investigated. However, we observed a significant decrease in post-dialysis serum ox-LDL levels with SHFVE-HD. Because of its molecular weight exceeding that of albumin, the clearance of ox-LDL was unlikely, suggesting that the observed decline could result from the antioxidant effects of vitamin E. Surprisingly, SHFVE-HD induced lower serum SOD activity levels after 15 min of dialysis initiation compared with MCO-HD, albeit without difference pre- and post-dialysis levels. As previous conflicting reports on erythrocyte [28] and serum SOD activity in HD [39], our study does not allow drawing firm conclusions about the effects of SHFVE-HD on this antioxidative enzyme.

ADMA is an endogenous inhibitor of endothelial nitric oxide synthase, which pre-dialysis serum concentration increases in HD patients and is an independent predictor of mortality and cardiovascular outcomes [40]. The present study confirms that compared with MCO-HD, SHFVE-HD provides lower ADMA pre-dialysis levels [36]. ADMA concentrations at 15 min and after dialysis were not measured, since because of its small size, this solute is easily removed by HD [3].

Transient reduction in peripheral blood leukocyte count, leucocyte activation, platelet activation, coagulation system activation and activation of the complement alternative pathway have been described as reliable markers of biocompatibility over the past decades [26]. Although dialyzer biocompatibility has improved with the use of synthetic membranes [15], we observed with SHFVE-HD a significant lower polymorphonuclear neutrophil and monocyte fluctuation rate at 15 min after dialysis initiation. This may suggest decreased monocyte activation with lower release of pro-inflammatory cytokines and lower activation of polymorphonuclear neutrophils with less oxidative stress. In addition, we found no difference between SHFVE-HD and MCO-HD on platelet activation, as assessed by platelet count fluctuation. Contrasting with our results, a small prospective study showed that a vitamin E–coated HF polysulfone dialyzer induced more stability in platelet fluctuation without difference in leucocyte fluctuation [41]. However, although early temporary intradialytic contact of blood with artificial materials can lead to leucocyte activation and the release of pro-inflammatory cytokines and ROS, the effect on long-term clinical outcomes remains unproven.

Finally, we found no difference in hemoglobin levels, transferrin saturation index, ferritin, reticulocyte hemoglobin content, ERI, ESA and iron dose between MCO-HD and SHFVE-HD. In a meta-analysis on HD with vitamin E–coated dialyzers, only partial effects on ERI and transferrin saturation index were reported [28].

The strength of our study is the evaluation of the middle-term effects of SHFVE-HD on uremic toxins and biocompatibility, based on a wide range of biomarkers. Limitations include the possible effect of treatment allocation before randomization, small sample size, lack of blinding, single-center design, short time study duration with focus on biomarkers only, calculation methods and lack of dialysate collection to determine the clearance of solutes. In addition, the assessment of dialyzer biocompatibility is a challenging process due to the methods used for evaluation and to patient-related clinical factors.

In conclusion, the present study indicates that SHFVE-HD achieves non-inferior beta2-microglobulin RR as compared with MCO-HD, with similar other MM RR. Therefore, it could be classified as a MCO dialyzer. In addition, SFHVE may provide additional benefits on biocompatibility parameters including inflammation, oxidative stress and leucocyte fluctuation. However, the long-term clinical benefits of this strategy remain to be investigated.

## Supplementary Material

sfaf106_Supplemental_File

## Data Availability

The research data are not publicly available. The data have been collected in a protected eCRF file and stored at the Centre de la recherche Clinique, CHU Poitiers. The data underlying this article will be shared on reasonable request to the corresponding author.
