# Supplementary material for: Effects of super high-flux vitamin E–coated and medium cut-off dialyzers on uremic toxins removal and biocompatibility: the E-FLUX randomized controlled study
Source: Clin Kidney J. 2025 Apr 11;18(5):sfaf106. doi: 10.1093/ckj/sfaf106 (PMC12059640; doi:10.1093/ckj/sfaf106)
Supplement: sfaf106_Supplemental_File [file sfaf106_supplemental_file.docx]

**Supplementary methods**.

Description of sampling and analysis

The following serum parameters were measured locally: urea, creatinine, CRP, serum amyloid A (SAA), ferritin, transferrin saturation index, lactate dehydrogenase, reticulocyte hemoglobin content, leukocytes, hemoglobin, hematocrit and platelets. Beta2-microgloblin (reference range: 0.8-2.2 mg/l) and myoglobin (reference range 10-90 μg/l) concentrations were measured by immunoturbidimetry (**Tina-quant, Roche Diagnostics GmbH, Mannheim).** Serum albumin (reference range: 35-52 g/l) was measured using the bromcresol green colorimetric test (**Roche Diagnostics GmbH, Mannheim)** and transthyretin (reference range: 0.2-0.4 mg/l) by nephelometry with a Siemens BNII analyzer (Siemens healthcare Diagnostics Products GmbH, Marburg, Germany).

According to the parameters evaluated, additional tests were performed pre-dialysis, pre and post-dialysis, or pre-dialysis, at 15 minutes after initiation and post-dialysis on serum obtained after immediate centrifugation for 10 min and stored at -80ºC. Levels of human interleukin 6 (IL-6), tumor necrosis factor-alpha (TNF-α), soluble tumor necrosis factor-alpha receptor 1 (sTNFR1), hepcidin and vascular endothelial growth factor (VEGF) were quantified using ELISA kit (R&D Systems Europe, Bio-Techne, Abingdon, UK). Elisa tests were also used to determine concentrations of Asymmetric dimethylarginine (ADMA) and malondialdehyde (MDA) (Novus biologicals Bio-Techne, Abingdon, UK), of superoxide dismutase (SOD) activity (SOD Assay Kit) (Cayman chemical, Ann Arbor, MI-USA) and of human oxidized low density lipoprotein (ox-LDL) (Mercodia, Uppsala, Sweden).

Prolactin was quantified using the Elecsys Prolactin II electrochemiluminescence (Roche Diagnostics GmbH, Mannhein Roche). Kappa and lambda serum free light chains (FLC) levels were quantified by nephelometry using the Freelite (The binding Site, Birmingham, UK) assay on a Siemens BN Prospect Analyzer (Siemens, Marburg Germany).

Calculations

Reduction ratios (RR) were calculated as follows: RR: ((Cpre-Cpost)/Cpre), where Cpre and Cpost are the pre and post treatment concentrations, respectively. Post treatment concentrations of MM (beta2-microglobulin, myoglobin, prolactin, alpha1-microglobulin, VEGF, kappa and lambda FLC) were corrected for hemoconcentration using a single-compartment kinetic model, as follows: Cpost corrected = Cpost/(1+ΔBW)/(0.2xBWpost), where ΔBW is the weight lost during the session and BWpost the body weight at the end of the session.

Markers of inflammation (IL-6, TNFα, TNFR1, Hepcidin), markers of oxidative stress (MDA, SOD activity, Ox-LDL), leucocyte and platelet counts were corrected for ultrafiltration-induced hemoconcentration according to Schneditz formula: hp=H1/H0*(100-H0)/(100-H1), where hp, H0 and H1 are hemoconcentration factor, pre-dialysis hematocrit and hematocrit at sampling time points (15 min after dialysis initiation and post-dialysis, respectively).

Whether or not the values were corrected for ultrafiltration-induced hemoconcentration, the significant results obtained in our study were similar.

Erythropoietin resistance index (ERI) was defined as the weekly dose of erythropoietin stimulating agents (ESA) per body weight divided by hemoglobin (Hb): (U/kg/g/dl/week) = weekly erythropoietin dose / (weight × Hemoglobin). All patients were treated with darbepoetin alpha in the study. ERI for darbepoetin alpha-treated patients was converted to an erythropoietin-equivalent value using a dose conversion factor of 200:1

Supplementary material table S1. Characteristics of high-flux dialysis membranes before randomization

| **Dialyzer**  **Number of patients (n=36)** | **Membrane polymer** | **Membrane type** | **Fiber inner diameter (μm)** | **Membrane area (m²)** | **Membrane wall thickness (μm)** | **UFC (ml/h/mmHg)** | **Beta2-m SC** | **Albumin SC** | **Sterilization** |
| --- | --- | --- | --- | --- | --- | --- | --- | --- | --- |
| **TS-2.1 SL™**  **n=10 (28%)** | **Polysulfone** | **High-flux** | **200** | **2.1** | **40** | **52** | **0.8** | **0.003** | **Gamma-Ray** |
| **V-20 HF™**  **n=12 (33%)** | **PES** | **High-flux** | **200** | **2** | **40** | **66** | **≥ 0.7** | **≤ 0.001** | **Gamma-Ray** |
| **Elisio**  **21H™**  **n=14 (39%)** | **Polynephron /PES** | **High-flux** | **200** | **2.1** | **40** | **82** | **0.8** | **0.002** | **Dry Gamma** |

Abbreviations: PAES/PVP: polyarylethersulfone/polyvinylpyrrolidone, PES: polyethersulfone, UFC : ultrafiltration coefficient, Beta2-m : beta2microglobulin, SC : Sieving coefficient

Supplementary material table S2. Baseline levels and reduction ratio of uremic toxins

|  | **All (n=36)** | **SHFVE-HD first (n=16)** | **MCO-HD first (n=20)** |
| --- | --- | --- | --- |
| Urea |  |  |  |
| - Pre-dialysis (mmol/L) | 21 (17-24) | 19 (17-21) | 22 (17-24) |
| - Post-dialysis (mmol/L) | 5 (3.7-6.2) | 4.2 (3.6-5.3) | 5.4 (4.8-6.4) |
| - RR (%) | 75±6.3 | 76±6.4 | 74±6.2 |
| Creatinine |  |  |  |
| - Pre-dialysis (μmol/L) | 659 (564-806) | 618 (485-786) | 684 (584-806) |
| - Post-dialysis (μmol/L) | 206 (156-304) | 204 (128-293) | 208 (172-304) |
| -RR (%) | 67±6.2 | 68±7.4 | 66±5.2 |
| spKT/V |  |  |  |
| Beta2-microglobulin (11.8 kDa)  - Pre-dialysis (mg/L)  - Post-dialysis (mg/L)  - RR (%) | 26 (22-29)  10 (6-12)  66±9.1 | 23 (19-28)  7 (5-10)  69±7 | 27 (25-30)  10 (7-13)  62±9.5 |
| Myoglobin (17 kDa)  - Pre-dialysis (µg/L)  - Post-dialysis (µg/L)  - RR (%) | 165 (119-230)  107 (63-143)  35±15 | 161 (100-195)  89 (50-129)  39±15 | 165 (121-255)  114 (86-177)  31±14.5 |
| Prolactin (23 kDa) **  - Pre-dialysis (ng/mL)  - Post-dialysis (ng/mL)  - RR (%) | 20 (14-35)  14 (10-28)  26±25 | 16 (11-28)  10 (9-15)  32±21 | 25 (18-39)  20 (14-31)  20±28 |
| Alpha1-microglobulin (30 kDa) ⃰ ⃰ ⃰ ⃰  - Pre-dialysis (µg/mL)  - Post-dialysis (ng/mL)  - RR (%) | 299±77  308±100  -3±20 | 293±89  299±108  -2±21 | 304±69  316±93  -3±20 |
| VEGF (43 kDa) ⃰ ⃰ ⃰ ⃰  - Pre-dialysis (pg/mL)  - Post-dialysis (pg/mL)  - RR (%) | 62 (37-128)  46 (27-87)  31±25 | 91 (38-140)  62 (28-102)  35±27 | 50 (35-118)  40 (27-60)  27±25 |
| Kappa FLC (22 kDa) *  - Pre-dialysis (mg/L)  - Post-dialysis (mg/L)  - RR (%) | 133±47  87±38  37±13 | 127±34  79±26  38±13 | 138±55  93±45  36±12 |
| Lambda FLC (45 kDa) ⃰ ⃰ ⃰  - Pre-dialysis (mg/L)  - Post-dialysis (mg/L)  - RR (%) | 107 (71-140)  96 (62-138)  4 (-2 to 16) | 75 (65-135)  75 (59-128)  1 (-0.4 to 18) | 111 (74-146)  102 (67-142)  3 (-3 to 14) |

Abbreviations: SHFVE (vitamin E-coated super High flux), MCO (medium cut-off), HD (Hemodialysis), spKt/V (single pool Kt/V), kDa (kiloDalton), VEGF (vascular endothelial growth factor), FLC (free light chains), RR (reduction ratio), SD (standard deviation), IQR (interquartile range). Quantitative data are expressed as mean ± SD or median (IQR). *: 1 missing data, **: 2 missing data, ⃰ ⃰ ⃰ 3 missing data, ⃰ ⃰ ⃰ ⃰ 4 missing data

Supplementary material table S3. Baseline levels of inflammatory parameters and markers of oxidative stress.

|  | **All (n=36)** | **SHFVE-HD first (n=16)** | **MCO-HD first (n=20)** |
| --- | --- | --- | --- |
| IL-6  -Pre-dialysis (pg/mL)  -After 15 min (pg/mL)  -Post-dialysis (pg/mL) | 0.7 (0.1-2.3)  0.8 (0.4-2.2)  0.8 (0.2-4.7) | 0.9 (0.2-16.5)  0.8 (0.3-17)  1 (0.3-21.5) | 0.6 (0.1-1.7)  0.8 (0.4-1.7)  0.7 (0-4.7) |
| TNF-α  -Pre-dialysis (pg/mL)  -After 15 min (pg/mL)  -Post-dialysis (pg/mL) | - 1. (0.1-1.8)   1 (0.1-5.4)  0.1 (0.1-0.1) | 0.6 (0.1-19.8)  3 (0.1-21)  0.1 (0.1-13.4) | 0.1 (0.1-0.1)  0.5 (0.1-2.6)  0.1 (0.1-0.1) |
| sTNFR1  -Pre-dialysis (ng/mL)  -After 15 min (ng/mL)  - Post-dialysis (ng/mL) | 14±3.9  15±4.2  13.6±4 | 12.3±3.3  13±3.4  11.7±3.3 | 15.3±3.9  16.5±4.2  15.1±3.9 |
| Hepcidin (2 to 3 kDa)  -Pre-dialysis (ng/mL)  -After 15 min (ng/mL)  -Post-dialysis (ng/m) | 102 (19-150)  80 (23-97)  72 (24-118) | 54 (12-127)  70 (9-103)  61 (8-99) | 115 (78-210)  83 (62-97)  88 (54-152) |
| Ox-LDL  -Pre-dialysis (U/L)  -After 15 min (U/L)  -Post-dialysis (U/L) | 56.4±21  53.1±18  59.3±18 | 56.3±22  52.5±22  56.5±19 | 56.6±21  53.6±16  61.5±18 |
| SOD activity ⃰  -Pre-dialysis (U/mL)  -After 15 min (U/mL)  -Post-dialysis (U/mL) | 4.6±2.1  4.6±2.1  3.5±1.7 | 4.4±2.5  4.3±2.4  3.4±2.3 | 4.8±1.9  4.8±1.9  3.6±1 |
| MDA  -Pre-dialysis (ng/mL)  -After 15 min (ng/mL)  -Post-dialysis (ng/mL) | 271 (169-411)  298 (178-538)  239 (152-499) | 264 (198-372)  198(234-480)  261 (173-455) | 321 (142-573)  314 (149-627)  190 (142-549) |
| ADMA |  |  |  |
| -Pre-dialysis (ng/mL) | 164 (118-262) | 132 (103-249) | 188 (139-337) |

Abbreviation: SHFVE (vitamin E-coated super High flux), MCO (medium cut-off), HD (Hemodialysis), IL-6 (interleukin 6), TNF-α (tumor necrosis factor alpha), sTNFR1 (soluble tumor necrosis factor receptor 1), Ox-LDL (oxidized low density lipoprotein), SOD (superoxide dismutase), MDA (malondihaldehyde), ADMA (asymmetric dimethylarginine), SD (standard deviation), IQR (interquartile range). Quantitative data are expressed as mean ± SD or median (IQR). * 2 missing data

Supplementary material table S4. Baseline routine biological parameters

|  | **All (n=36)** | **SHFVE-HD first (n=16)** | **MCO-HD first (n=20)** |
| --- | --- | --- | --- |
| Albumin |  |  |  |
| -Pre-dialysis (g/l) | 38±3.2 | 38.7±3.1 | 37.4±3.5 |
| -Post-dialysis (g/l) | 42±4.2 | 42.1±3.5 | 41.9±5.2 |
| Transthyretin (g/L) | 0.3±0.1 | 0.3±0.1 | 0.3±0.1 |
| nPCR | 1±0.2 | 0.9±0.2 | 1±0.2 |
| CRP (mg/L) | 3.5 (1-10) | 2.5 (1-5.5) | 6.5 (1.5-14.5) |
| SAA (mg/L) | 11 (5-18) | 8 (3-13) | 16 (9-36) |
| Hemoglobin (g/dL) | 11.4±1.2 | 11.6±1.3 | 11.2±1.1 |
| Ferritin (µg/L) | 397 (255-521) | 341 (152-488) | 418 (349-538) |
| Transferin saturation index (%) | 24.4±10.9 | 24±9.7 | 25.1±12.5 |
| Ret-he (pg) | 31.7±3.8 | 32.4±4.5 | 31.2±3.1 |
| LDH (UI/l) | 198 (176-224) | 198 (178-220) | 197 (170-225) |

Abbreviations: SHFVE (vitamin E-coated super High flux), MCO (medium cut-off), HD (Hemodialysis), nPCR (normalized protein catabolic rate), CRP (C-reactive protein), SAA (serum amyloid A), Ret-he (hemoglobin content of reticulocytes), LDH (lactate dehydrogenase), SD (standard deviation), IQR (interquartile range).Quantitative data are expressed as mean ± SD or median (IQR).

Supplementary material table S5. Baseline leucocyte and platelet fluctuation

|  | **All (n=36)** | **SHFVE-HD first (n=16)** | **MCO-HD first (n=20)** |
| --- | --- | --- | --- |
| Leucocyte |  |  |  |
| -Pre-dialysis (10^9^ cells/L) | 5.7 (4.6-6.5) | 5.6 (4.4-6.4) | 5.8 (4.9-6.6) |
| -After 15 min (10^9^ cells/L) | 5.5 (4.3-6.1) | 5.2 (4.2-6) | 5.6 (4.4-6.2) |
| -Post-dialysis (10^9^ cells/L) | 5.3 (4.4-6.6) | 5.1 (3.9-6.6) | 5.4 (4.8-6.7) |
| -RV (%) after 15 min | -8.1 (-14.8 to -1.6) | -7.5 (-15.6 to -2.0) | -8.3 (-11.3 to 0.0) |
| -RV (%) after 240 min | -7.4 (-13.1 to 11.7) | -8.4 (-16.2 to 9.5) | -4.6 (-12.3 to 12.0) |
| Polymorphonuclear neutrophil |  |  |  |
| -Pre-dialysis (10^9^ cells/L) | 3.8±1.4 | 3.7±1 | 3.9±1.8 |
| -After 15 min (10^9^ cells/L) | 3.6±1.5 | 3.5±0.9 | 3.6±1.9 |
| -Post-dialysis (10^9^ cells/L) | 3.7±1.6 | 3.5±1.1 | 3.9±1.9 |
| -RV (%) after 15 min | -4.7 (-11.7 to 0.6) | -7.1 (-14.4 to -0.3) | -2.7 (-9.0 to 0.6) |
| -RV (%) after 240 min | -5.6 (-15.5 to 12.2) | -9.2 (-17.7 to 9.1) | 3.3 (-14.5 to 12.2) |
| Monocyte |  |  |  |
| -Pre-dialysis (10^9^ cells/L) | 0.6±0.2 | 0.6±0.2 | 0.6±0.3 |
| -After 15 min (10^9^ cells/L) | 0.5±0.2 | 0.5±0.2 | 0.4±0.2 |
| -Post-dialysis (10^9^ cells/L) | 0.6±0.2 | 0.6±0.2 | 0.5±0.1 |
| -RV (%) after 15 min | -22.4 (-36.1 to -15.0) | -20.7 (-33.0 to -12.8) | -24.4 (-36.1 to -15.8) |
| -RV (%) after 240 min | -9.8 (-20.2 to 10.4) | -10.8 (-20.1 to 1.8) | -9.8 (-20.2 to 16.7) |
| Platelet  -Pre-dialysis (10^9^ cells/L) | 189 (148-222) | 191 (166-201) | 178 (137-236) |
| -After 15 min (10^9^ cells/L) | 176 (133-210) | 177 (150-199) | 170 (133-234) |
| -Post-dialysis (10^9^ cells/L) | 188 (141-228) | 185 (162-217) | 198 (135-240) |
| -RV (%) after 15 min | -2.8 (-7.1 to 1.9) | -3.2 (-9.7 to 0.5) | -2.8 (-6.0 to 4.3) |
| -RV (%) after 240 min | 1.0 (-6.5 to 7.8) | 1.0 (-5.0 to 5.2) | 0.0 (-7.2 to 18.2) |

Abbreviation: SHFVE (vitamin E-coated super High flux), MCO (medium cut-off), HD (Hemodialysis), RV (relative variation), SD (standard deviation), IQR (interquartile range). Quantitative data are expressed as mean ± SD or median (IQR).
